# Algae associated with coral degradation affects risk assessment in coral reef fishes

**DOI:** 10.1038/s41598-017-17197-1

**Published:** 2017-12-05

**Authors:** Mark I. McCormick, Randall P. Barry, Bridie J. M. Allan

**Affiliations:** 1 ARC Centre of Excellence for Coral Reef Studies, and Department of Marine Biology and Aquaculture, James Cook University, Townsville, Queensland 4811 Australia; 2 Present Address: Institute of Marine Research, Bergen, Norway

## Abstract

Habitat degradation alters the chemical landscape through which information about community dynamics is transmitted. Olfactory information is crucial for risk assessment in aquatic organisms as predators release odours when they capture prey that lead to an alarm response in conspecific prey. Recent studies show some coral reef fishes are unable to use alarm odours when surrounded by dead-degraded coral. Our study examines the spatial and temporal dynamics of this alarm odour-nullifying effect, and which substratum types may be responsible. Field experiments showed that settlement-stage damselfish were not able to detect alarm odours within 2 m downcurrent of degraded coral, and that the antipredator response was re-established 20–40 min after transferral to live coral. Laboratory experiments indicate that the chemicals from common components of the degraded habitats, the cyanobacteria, *Okeania* sp., and diatom, *Pseudo-nitzschia* sp.prevented an alarm odour response. The same nullifying effect was found for the common red algae, *Galaxauria robusta*, suggesting that the problem is of a broader nature than previously realised. Those fish species best able to compensate for a lack of olfactory risk information at key times will be those potentially most resilient to the effects of coral degradation that operate through this mechanism.

## Introduction

Habitat modification of wilderness areas is a key cause of global losses in biodiversity^1,2^. Causes of this biodiversity loss include overexploitation of habitats and animals, reduced water quality, global warming and ocean acidification. In the marine environment over the last four decades marine vertebrates and fishes have declined in abundance by 22 and 38% respectively^3,4^. While the mechanisms underpinning the loss of species that comes with the wholesale destruction of a habitat are clear (e.g.,^5^), most habitat change involves a less complete degradation of habitat quality. This degradation of the resource provided by the habitat leads to a change in the balance of population and community processes that influence population replenishment, maintenance, and species coexistence (e.g.,^6^). Conservation ecologists can list many of the processes that are likely to be the drivers underlying community change^5,7^, which include the increased vulnerability to invasions^8^, parasites^9^ and predators^10^, reduction of prey^11^, and alterations of biophysical conditions^12^. To date, there are few detailed studies of the mechanisms that promote community change when habitats degrade, particularly in marine environments.

Coral reefs represent one of the worlds’ most biologically diverse ecosystems, but one that is particularly vulnerable to changes in environmental parameters because the major habitat-forming hard corals live very close to their thermal tolerance limits^13^. The world’s largest coral reef, the Great Barrier Reef (GBR) off Australia’s east coast, has lost 40% of live coral cover between 1990 and 2010^14,15^. Recent aerial surveys documented a major coral bleaching event during early 2016 with 50% of the reefs surveyed over the whole of the 2000 km of the GBR having >60% bleaching^16^, with another major episode occurring in early 2017 affecting the middle half of the GBR (Hughes unpublished data). Bleaching occurs when the symbiotic algae that provide most of the corals’ nutrition are expelled from the host. If unfavourable conditions persist coral death will follow resulting in degradation of coral tissue and structure^17^.

Recent research has found that some coral reef fishes lose the ability to assess threats through the use of alarm odours when in a habitat dominated by degraded coral^18–21^. Alarm odours are usually reliable indicators of a nearby threat because they are only released through damage of the epidermis of a conspecific^22^. They also play a central role in the identification and cataloguing of novel predators in addition to updating information on current threats^22,23^. So strong is this Pavlovian-style learning mechanism that fish can learn the identity of a predator from a one-off pairing of a conspecific alarm odour with a predator odour^24^. Indeed this mechanism is so basal to their learning framework that fishes can be trained to recognise unnatural stimuli as a threat, such as a red-light^25^, a black disc^26^ or lemon juice^27^.

Disruption to this important threat-mediated mechanism appears to occur when alarm odours interact with chemicals associated with dead-degraded coral^18^, although the mechanism underpinning this chemical alteration is unclear. Whatever the chemical mechanism, experiments have shown a reduced efficiency of alarm odours that reduces the ability of fish to learn the identity of new dangers^19^ and develop a general risk-averse response to novel cues (i.e., neophobia,^28^). This means that fishes who live on dead-degraded coral, or even those that are closely surrounded by dead coral but are actually living in association with live coral, are likely to be more active, feed more and stray further from shelter^29^. This scenario makes them more susceptible to predation, and fish under the influence of chemicals from dead-degraded coral sustain higher mortality during the critical early juvenile mortality-bottleneck^29–32^. Out of the seven species of damselfish tested to date for this phenomenon, the alarm odour nullifying effect has been found to influence the Ambon damselfish (*Pomacentrus amboinensis*), plus two other damselfish species (blue chromis, *Chromis* sp.and the lemon damsel *P*. *moluccensis*) that associate with live coral^19,20^.

To date, tests of the effects of the chemistry from dead-degraded coral habitat on the utility of fish alarm odours have been undertaken at very small spatial scales, confined within aquaria or in the field where the distance between degraded habitat and the focal fish has been less than one metre. To understand the broader ramifications of this potentially important process to population dynamics, assessments must be made of the spatial extent of the alarm odour-nullifying effect and the environmental factors, such as current strength, that may affect it. The provision of chemical information in both water and air depends strongly on the velocity and stability of the medium that it is carried by, as the movement of odours is largely a passive process^33^. This means that for a juvenile fish whose home range is often less than one square metre^34^, the chemistry of the water passing will depend on the composition and spatial arrangement of the habitat in the local vicinity. Given that most reefs are comprised of a mosaic of benthic habitat types (e.g.live hard coral, soft coral, dead-degraded corals, algae, sponges, ascidians etc), there may be periods when fish are exposed to water that has recently passed over degraded habitat, followed by periods of water influenced by less degraded habitats. Moreover, because the strength of flow over shallow reefs changes with tidal state and wind conditions, the concentration of chemicals from the local environment may also change. Whether these small-scale changes in local hydrology are important for risk assessment will depend on the whether or not the alarm odour-nullifying effect is reversible on a relevant time scale. Thus, key to understanding the importance of the alarm odour-nullifying effect is determining the spatial and temporal scale of the effect; how far away does a fish need to be before it is not affected by the chemicals that are emitted from dead-degraded habitat, and is the effect reversible?

Dead-degraded coral is itself a living habitat made up of a diverse array of bacteria, invertebrates and plants, which may be important components in producing the chemicals that leads to alarm odour alteration. As live coral dies, it undergoes a successional series involving bacteria, algae and invertebrates (e.g.,^35,36^) that modify and erode the coral skeleton. Indeed, it can be the blue-green algae that leads to the death of stressed corals through coral disease^37^. These rapidly growing filamentous cyanobacteria, together with benthic diatoms, are common elements of the benthic assemblage on degraded substrates^38^. Moreover, both cyanobateria and diatoms are known to metabolise and sequester a broad variety of secondary compounds that can be toxic to some vertebrates^39^, and sporadically occur in dense blooms^40,41^. Bacteria within the genus *Okeania* (previously grouped with *Lyngbya*) are a particularly common filamentous cyanobacteria in subtropical and tropical marine environments and are known to be chemically rich^42^, with species within the genus producing toxic metabolites^39,43^. It is possible that these bacteria, together with other components of the benthic flora, may be producing chemicals that lead to the alteration of the alarm odours found in the vicinity of dead-degraded coral.

The present study experimentally examined the spatial and temporal scale of the alarm odour-nullifying effect from dead-degraded coral in a natural setting for a juvenile fish, the Ambon damselfish *Pomacentrus amboinensis* (Pomacentridae). Specifically, we examined the effect of distance from dead-degraded coral on the response intensity of fish to alarm odour. A second field experiment determined whether the detrimental effect on alarm odour detection for these fish was permanent or reversible. The last part of the study involved a laboratory experiment that examined which components of the dead-degraded coral community cause the dramatic loss in the efficacy of alarm odours. The alarm odour responses of juvenile Ambon damselfish were assessed in the presence of water that has passed over five substratum types: live healthy coral, dead-degraded coral, dead clean coral skeleton, scrubbed dead-degraded coral, the cyanobacteria *Okeania*, and a benthic diatom *Pseudo-nitzschia* sp. The latter organisms are commonly found on dead coral at the study location. To determine whether the alarm odour-nullifying effect was unique to these benthic organisms, two other common substratum types were also included for comparison: the brown algae *Padina* sp.and the red algae *Galaxaura rugosa*, both of which can cover substantial portions of the shallow reef in tropical waters.

## Methods

### Study species and collection

The three studies were conducted at Lizard Island Research Station (14° 40′ S, 145° 28′ E) and fringing reef, on the northern Great Barrier Reef, Australia, during October–December 2016. The Ambon damselfish, *Pomacentrus amboinensis*, is a common fish within coral reef fish communities of the Indo-Pacific (especially on the Great Barrier Reef). Adults are protogynous and form lose social groups, which are found in highest densities in shallow areas with a mixture of sand, rubble and live hard coral^44^. Juveniles settle from the larval phase after 15–23 d (at about 10–12 mm SL, standard length) to a broad range of habitats including live coral (70% of settlers), dead coral (20%) and rubble (10%)^45^. At this stage, most damselfish species are strongly site attached and tagging studies suggest they do not move more than 1–2 m from their settlement site for the first few month after settlement^45,46^. At the end of their larval stage, fish larvae were collected at night using light traps^47^, returned to the laboratory and sorted by species before their transfer to flow-through seawater 35-L tanks. Light traps were moored at least 30 m from the nearest reef edge. Fish caught had not yet experienced the fish community on the benthic coral reef habitat and were unexperienced with reef-associated predators^48^. Fish collected in this way were used within 4 days of capture and were the source of fish for all field and laboratory experiments. Previous research on the Ambon damselfish has found that the newly-settled fish have an innate anti-predatory response to damage-released chemical cues from the skin of conspecifics both in the field and laboratory^18^. This reaction is typified by reduced foraging and activity, and increased shelter use, which is similar to the reaction shown in many other damselfishes^48,49^.

**Figure 1:**
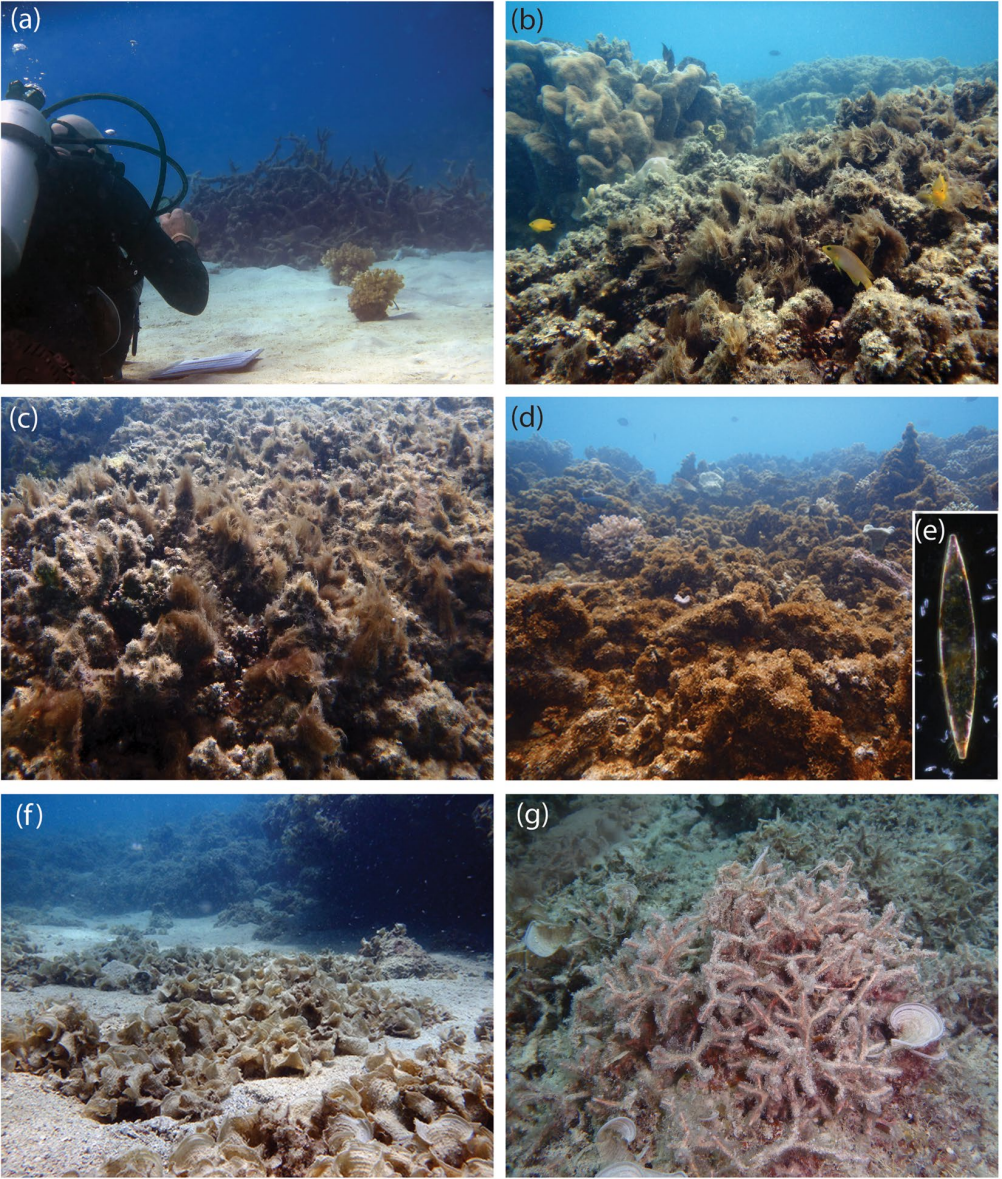
Habitat types under study. (**a**) Patches of live healthy *Pocillopora damicornis* on a shallow sand flat in front of a large bed of dead-degraded coral, with a diver observing the behaviour of a juvenile fish with the aid of a magnifying glass. (**b**) Degraded reef with a mixture of algal/bacterial species. (**c**) An area of dead coral covered with *Okeania* sp. cyanobacteria. (**d**) An area of dead coral covered with turfing algae covered with a diatom bloom of *Pseudo-nitzschia* sp. (**e**) The diatom, *Pseudo-nitzschia* sp.isolated from the bloom. (**f**) A sand gutter filled with the brown algae, *Padina* sp. (**g**) The red algae, *Galaxaura rugosa*, (surrounded by some *Padina* sp.). Photograph credits M. McCormick.

Live coral in the field and laboratory experiments refers to live healthy *Pocillopora damicornis*, which is a common bushy hard coral around the Lizard Island fringing reef and is a common nursery habitat for reef fishes. Dead-degraded coral refers to the same coral species, but in its dead form, covered with a mixture of bacteria, algae and invertebrates (Fig. 1).

### Field manipulations

#### Effect of distance from degraded coral on risk assessment

The field experiment involved assessing the behavioural response of juvenile Ambon damselfish before and after being exposed to one of two olfactory cues, once they had been habituated to small live coral patch reefs at 4 distances down-current of a bank of dead-degraded coral: 0.1 m, 0.5 m, 2 m and 5 m. Two sites were used for the experiment at the edge of a reef with a unidirectional flow of water (mean ± SD, 0.18 ± 0.06 m/s) parallel to the reef edge and perpendicular to natural projections of dead-degraded coral projecting from the reef edge into the adjacent sandflat (Fig. 1a). At each site current speed was estimated 3 times prior to starting each trial, allowing a current speed to be assigned to each replicate. Current was estimated by timing the progress of a neutrally buoyant object over a 2 m distance. Current speed ranged between 0.03 to 0.33 m/s. Data were collected during 13 days spread over a month between late-October and late-November 2016.

Fish were placed individually into 1-L plastic bags and transported to the experimental sites in a darkened 60-L container of seawater to minimize the stress associated with transport. Fish were then released individually onto patch reefs (25 × 20 × 20 cm) consisting of live healthy *Pocillopora damicornis*. All resident fishes and/or mobile invertebrates were removed from these patches prior to the introduction of the focal fish. Fish were given between 20 and 60 min to habituate to the patch reef, with treatments randomly allocated with respect to acclimation period. Fish have been found to start feeding within 30 s of release^50^, suggesting that they rapidly recover from the stress of transportation and release once placed on a natural habitat patch.

**Figure 2:**
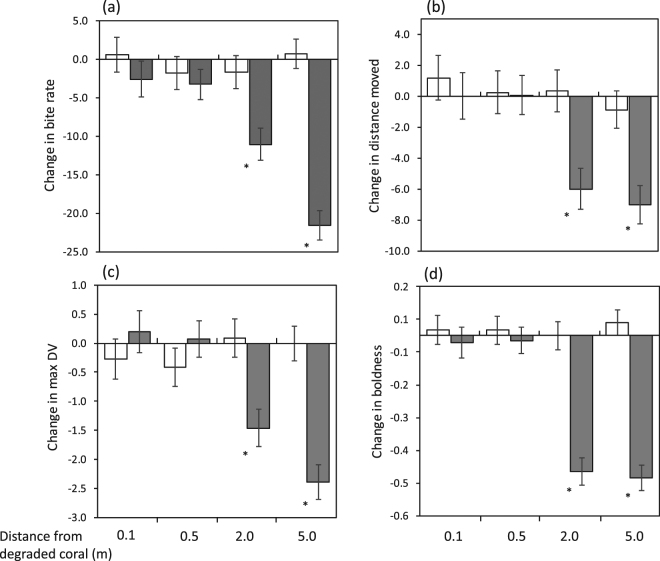
Effect of distance down-current from degraded coral on the detection of chemical alarm cues by *Pomacentrus amboinensis* on live coral patch reefs. Fish were exposed to either saltwater (white) or alarm odour (grey) by a tube up-current of the patch reef and their change in behaviour recorded over 3 min (before and after cue) as: (**a**) bite rates, (**b**) total distance moved, (**c**) maximum distance ventured from shelter, (**d**) boldness index (expressed as a continuous scale from 0 = shy to 3 = bold, see text for details). Asterisks represent significant differences between SW and alarm odour response by planned comparison. N (left to right) were: 11, 12, 12, 15, 10, 14, 13, 15.

The innate antipredator response of fish to conspecific alarm odours was used to assess whether distance from dead-degraded coral affected risk assessment. Other replicate fish were exposed to seawater to control for the introduction of an odour onto the patch. Fish were only used once. This gave a 2 (Cues) × 4 (Distances) ANOVA design with 10 to 15 replicates (see Fig. 2 legend for specific replicates).

To prepare the damage-released olfactory cues underwater, a small fish (a recently-settled juvenile Ambon damselfish) was placed into a 75 × 125 mm clip-sealed bag filled with ~100 ml of ambient sea water from near the patch reef of the focal fish. Fish were euthanized by a quick blow to the brain case and macerated within the bag. Fish used as cue donors were of similar size to the focal fish (12–14 mm standard length), with one donor fish used per replicate. The plastic bag was then pierced with a 60 ml syringe to obtain a sample that represented the alarm odour treatment. The alarm odour sample was clear and colourless. The cue was delivered onto the patch reefs by a 2 m long plastic tube positioned up-current of the patch. Cues (60 ml) were slowly injected via a syringe, and then flushed with a further 60 ml of ambient seawater. Both the seawater (control) and alarm odour syringes were prepared prior to the initiation of a trial and placed in a catch bag close to the single observer (MIM). Syringes used in a trial were a random choice of the two and the identity of the cue used was unknown to the observer prior to the completion of a pair of trials (i.e., trials were blind). In addition, a slip of underwater paper was used to conceal previous trials on the data transcription board to prevent reference to the ‘before’ assessment data during the ‘after’ cue behavioural assessment.

#### Is the effect reversible?

To determine whether the alarm odour-nullifying effect that occurs in association with dead-degraded coral was reversible, light trap caught Ambon damselfish were tested for an alarm odour response on dead-degraded coral, and then transferred to a live coral patch and retested 20 to 40 min later. Fish were carefully transferred between patches with the aid of a hand-net. Fish were tested in exactly the same way as in the previous field experiment, with exposure of random habituated fish to either an alarm odour or a seawater control, with their behaviour assessed for 3 min both before and after the introduction of a cue through a tube.

#### Behavioural assessment

Twenty to 60 min after release onto a patch reef, a single observer (MIM) assessed the behaviour and space use of the fish using a well-established behavioural protocol (e.g.,^31^). In brief, fish behavior was assessed over a 3 min period by an observer that was ~1.5 m away from the patch reef with the aid of a magnifying glass. Four aspects of activity and space use were assessed: (i) bite rate; (ii) total distance moved (estimated from the known length of each reef); (iii) distance ventured from the habitat patch (categorized as % of time spent within 0, 2, 5 or 10 cm away from the patch); (iv) relative level of boldness. The behavioural axis of boldness through to shyness (boldness index) in part represents a risk-sensitivity index and was assessed using a continuous scale between 0 and 3 where: 0 is hiding in hole and seldom emerging; 1 is retreating to hole when scared and taking more than 5 sec to re-emerge, weakly or tentatively striking at food; 2 is shying to shelter of patch when scared but quickly emerging, purposeful strikes at food; and 3 is not hiding when scared, exploring around the coral patch, and striking aggressively at food^31^. This boldness index has been shown to be repeatable (e.g., repeatability values of ~0.5 over a 2 h period^51,52^). Our previous studies have shown that the behavior and space use by recently settled damselfish is remarkably consistent over time periods up to 5 days and that 3 min is a sufficiently long enough time period to quantify this behaviour^45,52,53^. Mean distance ventured from the patch was calculated as the cumulative proportion of the time spent at different distance from shelter over the 3 min sampling period. Behavioural differences between the pre- and post-cue period were used as the measure of the fish’s response to the cue. Reductions in feeding and space use are both well-established antipredator responses^22^.

### Laboratory studies

#### Effect of water source on alarm response

To determine which component of the dead-degraded coral community may be responsible for the alteration of the chemical alarm odour response, laboratory trials were undertaken with Ambon damselfish to determine its response to alarm odour in seawater that had been passed over one of 6 different substrata or benthic components. These substrata were: Live healthy *Poc*. *damicornis*; sun-bleached coral skeleton; dead-degraded coral covered with a typical algae and invertebrate assemblage; dead-degraded coral scrubbed under flowing seawater with a small brush, removing most of the macroalgae; the filamentous cyanobacteria *Okeania* sp.; a blooming *Pseudo-nitzschia* sp. diatom that attaches to the epilithic algal matrix to form a bronze-orange turf; the brown macroalga *Padina* sp.; and the spongy net-like red algae *Galaxaura rugosa* (Fig. 1). Each tank contained an amount of substrata (amount per unit area) equivalent to places on the reef where the substrata were abundant (examples pictured in Fig. 1).

Fish were preconditioned with seawater that had passed through one of four 35 L header tanks containing the treatment substratum and into one 35 L aquaria containing shelter and 10 to 20 juvenile Ambon damselfish (giving 4 tanks per treatment) for a 48 h period.

Following the conditioning phase, fish were moved individually into 15 L plastic aquaria containing sand, a molded plastic branched coral model (15 cm high) shelter, and an air stone, to which was attached a 1.5 m long injection hose. Each test tank received flow-through water from a header tank containing one of the treatment substrata, as previously described, but with the header tank flow being divided into 7 testing tanks. Each test tank thus received water at a rate of ~0.7 L/min (one tank turnover every 25 min). Three sides of the tanks were covered in black plastic to prevent interactions with neighbouring fish. The fish were left to acclimate overnight and were tested the following day.

The testing phase followed an established protocol^54^. Two mL of an *Artemia* solution (~80 *Artemia*/mL) was initially added followed by a 2-min pre-stimulus observation period. Variables recorded were the number of feeding strikes, and the number of lines crossed of a 4 × 4 cm grid drawn on the side of the aquarium. After this baseline observation period, we injected 10 mL of a solution of conspecific alarm odour or a 10 mL of seawater (SW) as an injection control, followed by 2 ml of *Artemia* and this was flushed slowly into the tank with 30 ml seawater. Fish behaviours (feeding strikes and line-crosses) were recorded after the cue injection for another 3 min period. Similarly to the field studies above, behavioural differences between the pre- and post-cue period were used as the measure of the fish’s response to the cues.

Conspecific alarm odours were prepared by euthanizing 3 conspecific donors via cold shock, and making nine cuts on either side of their bodies. The bodies were then rinsed with 15 mL of seawater and this 15 mL solution was used fresh within 15 min of preparation. This concentration has previously been shown to elicit overt antipredator responses in damselfishes^55^. The observer was blind to the treatment and the order of treatment was randomized.

### Statistical analyses

To determine whether the distance down-current from banks of dead-degraded coral (factor: Distance) affected the reaction of Ambon damselfish juveniles to seawater (SW) or alarm odour (factor: Cues), a two-factor MANOVA (factors: Distance, Cues) was conducted on the change in behaviour between 3 min behavioural assessments before and after the addition of cues. Bite rate, total distance moved, maximum distance ventured and boldness index were included in the analysis. Data were checked for normality and homogeneity of variance using residual analysis and was found to meet assumptions. Pillai’s trace was used as the test statistic. To further explore the nature of differences found by MANOVA, two-factor (fixed) ANOVA’s were undertaken on individual variables (Type III SS), followed by planned comparisons testing between SW and alarm odour treatments within each distance level. Initially linear mixed effects models were run on each of the four behavioural variables incorporating site as a random effect and current speed as a covariate. These preliminary analyses found site and current to be non-significant contributors to variance so they were excluded from subsequent analyses. Analyses were performed with Statistica (Dell, version 13, variance estimation and precision routine).

To examine whether the detrimental effects of dead-degraded coral on alarm odour responses were reversible, we employed a repeated measures ANOVA that accounted for the repeated measures on each fish. Here the focal fish’s behavioural response to one of two Cues (SW or alarm odour) was first tested on dead-degraded coral and later on live coral, representing two levels of coral state (State: dead coral, live coral). The variables tested were bite rate, distance moved, mean distance ventured from shelter and boldness index. If the interaction between coral State and Cue were significant, then the nature of the differences were determined using Tukey’s HSD post-hoc means comparisons. Assumptions of homogeneity of variance and normality were examined using residual analysis. There was no assumption of sphericity because there are only two repeated samplings of each individual fish.

To determine whether the habitat type that the seawater have passed (Source) affected the way that the Ambon damselfish responded to alarm odours, a two-factor ANOVA was performed with the factors Source (one of eight substrata in header tanks) crossed with Cue (SW or alarm odour) for the variables feeding rate and line-crosses. To determine the source of the interactions found, planned comparisons were used to test for differences between SW and water source within each substratum type in the header tanks. Effect sizes, expressed as partial eta squared (ηp^2^) of the paired tests, are also given. Assumptions of homogeneity of variance and normality were examined using residual analysis.

## Results

### Effect of distance from degraded coral on risk assessment

Distance down-current from a bank of dead-degraded coral affected the ability of the Ambon damselfish to use alarm odours to assess risk (MANOVA, Distance x Cue interaction, Pillai’s Trace = 0.52, F_12,279_ = 4.84, p < 0.0001). Prevalent current speed at the time of the behavioural observations did not alter the effects of Distance, Cue or their interaction (covariate, p = 0.72).

Bite rate was affected by the cue, but whether the response to SW differed to the response to alarm odour depended on the distance from the bank of degraded coral (Cue x Distance interaction: F_3,94_ = 11.03, p < 0.0001). There was no difference in bite rate between cues at 0.1 and 0.5 m, but the bite rates of the alarm odour-exposed fish were reduced by 50% at 2 and 5 m from the source of the degraded water (Fig. 2a) suggesting that the efficacy of alarm odour to inform risk was restored due to increased distance from degraded coral. Change in the total distance moved (Fig. 2b), change in maximum distance ventured from the edge of the patch (Fig. 2c), and change in boldness (Fig. 2d) all showed the same patterns as displayed for bite rate (Table 1), with the reaction to alarm odour only occurring once at least 2 m from the bank of dead coral.

**Table 1:** Comparison of four behavioural traits of juvenile Ambon damselfish placed on patches of live coral positioned at four distances (0.1, 0.5, 2 and 5 m) from banks of dead-degraded coral and exposed to one of two olfactory cues (seawater, alarm odour). Results are from ANOVA’s on individual variables.

Source	Bite rate		Distance moved		Maximum DV		Boldness index	
	F	p	F	p	F	p	F	p
Distance(3,94)	8.36	<**0**.**0001**	5.65	**0**.**001**	5.57	**0**.**001**	15.45	<**0**.**0001**
Cue(1,94)	36.84	<**0**.**0001**	13.30	**0**.**0004**	10.44	**0**.**002**	64.48	<**0**.**0001**
Distance x Cue(3,94)	11.03	<**0**.**0001**	2.98	**0**.**035**	10.44	<**0**.**0001**	16.30	<**0**.**0001**

### Is the effect reversible?

**Figure 3:**
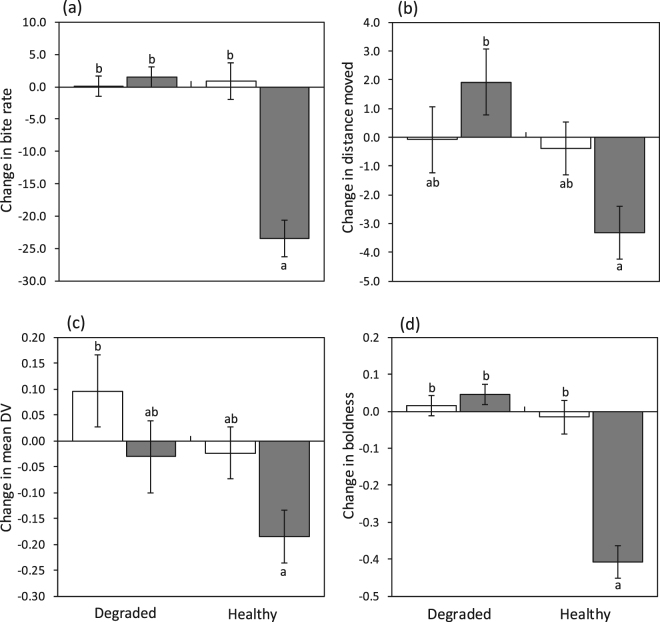
Change in bite rate (**a**), total distance moved (**b**), mean distance ventured (**c**) and boldness index (**d**) (means ± SE) for the Ambon (*Pomacentrus amboinensis*) exposed to either seawater (white) or conspecific alarm odour (grey) while residing on firstly a patch of dead-degraded coral, and then a patch of healthy live coral for 20–40 min. Change is calculated as ‘after’ subtracted from ‘before’ the injection of the cue. Lowercase letters above error bars represent Tukey’s HSD means comparisons. N (from left) = 15, 21, 15, 21.

While the Ambon damselfish showed no response to conspecific alarm odours when on dead-degraded coral, they responded with a significant decrease in bite rate (Fig. 3a, Table 2) and boldness index (Fig. 3d, Table 2) when on live coral (as indicated by the significant interactions: Cue x State). This suggests that the 20–40 min time period spent habituating on the live coral prior to being retested was sufficient for them to recover their ability to identify their alarm odour as a threat. Total distance moved and mean distance ventured (DV) had similar trends to bite rate, but showed more variability and non-significant interactions (p = 0.053 and p = 0.075 respectively; Fig. 3c,d, Table 2). While these behaviours displayed a more variable response to alarm odour when fish were on dead coral, both species showed much lower distances moved and ventured when exposed to alarm odours on live coral (Table 2, Fig. 3b,c).

**Table 2:** Comparison of behavioural variables for juvenile Ambon damselfish that were placed onto a dead coral patch and tested for a response to either seawater or conspecific alarm odours (Cue), and then transferred to a live coral patch and tested again for their response to the same cue. ‘State’ is a repeated measures variable that accounts for any change in behaviour of individuals as they are transferred between the two coral health states. Degrees of freedom are given in brackets.

Source	Bite rate		Distance moved		Mean DV		Boldness index	
	F	p	F	p	F	p	F	p
Cue(1,24)	23.26	<**0**.**0001**	0.30	0.587	6.18	**0**.**020**	23.42	<**0**.**0001**
State(1,24)	28.83	<**0**.**0001**	5.26	**0**.**031**	4.62	**0**.**042**	41.63	<**0**.**0001**
Cue x State(1,24)	33.03	<**0**.**0001**	4.16	0.053	0.07	0.075	31.73	<**0**.**0001**

### Effect of water source on alarm response

**Figure 4:**
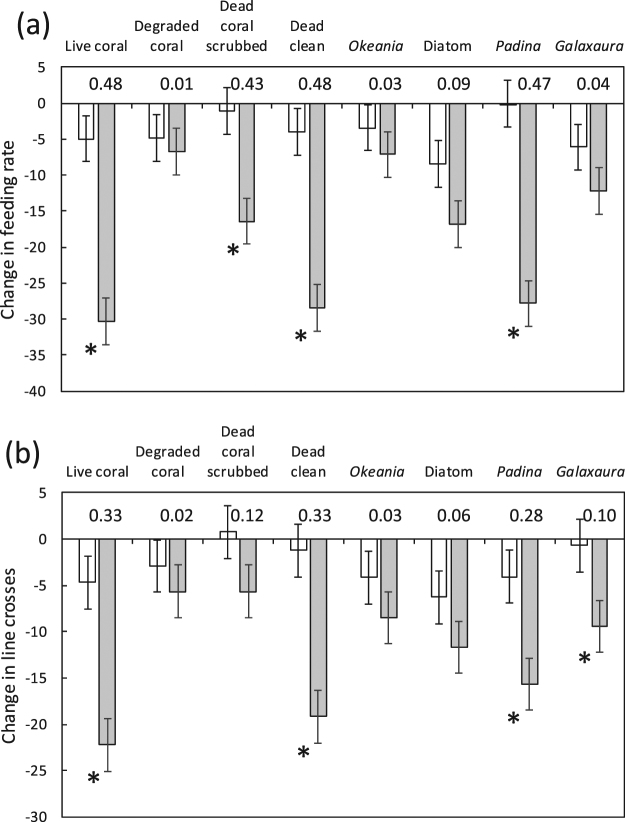
Habitat type affects risk assessment. Influence of the habitat type within the header tanks that supply the observation aquaria on the response of *Pomacentrus amboinensis* to either saltwater (white) or alarm odours (grey). (**a**) The mean number of feeding strikes (±SE) and (**b**) line crosses as a measure of activity are given. Asterisks represent statistically significant planned comparisons (p < 0.05), and numbers above the x-axis are the effect sizes (ηp^2^) of the paired tests. n = 17.

The substratum within the header tank (i.e.water source) affected whether the Ambon damselfish responded to alarm odours (feeding rate, Source x Cue: F_7,256_ = 5.407, p < 0.001; line crosses, Source x Cue: F_7,256_ = 2.146, p = 0.040; Fig. 4a,b). When fish were exposed to water that had passed over live coral, dead-scrubbed coral, dead clean coral or *Padina* there was a significant reduction in the feeding and activity rates (except dead-scrubbed coral) in response to the introduction of alarm odour compared to when seawater was introduced alone (Fig. 4a,b, planned comparisons). In contrast, there was no significant change in feeding or activity rate when fish were exposed to water that had passed over dead-degraded coral, the filamentous cyanobacteria *Okeania*, or the *Pseudo-nitzschia* diatoms (Fig. 4a,b, planned comparisons), suggesting a loss of alarm odour efficacy. When the red algae, *Galaxaura*, was in the header tanks there was no significant reduction in feeding rate by the damselfish when exposed to alarm odours (Fig. 4a), but there was a significant reduction in line crosses (Fig. 4b), suggesting that the substratum partially lead to impaired antipredator performances. This suggests that the algal components that cover the dead-degraded calcium carbonate skeleton produced chemicals that may nullify the effects of the alarm odour for the Ambon damselfish, as does the cyanobacteria, diatom and the red algae tested in the study.

## Discussion

This study emphasises that the environment within which the animal lives is central to the cues received to inform the behavioural trade-offs that balance risk and vigilance against other fitness promoting activities. Recent research emphasises the importance of water-borne chemicals for aquatic organisms, and that chemical information can play a central role in key behavioural decisions associated with antipredator responses^56,57^. Chemicals that are passively or actively released into the surrounding water contain ecologically relevant information that includes reproductive state^58,59^, stress^60^, disturbance^61^, habitat suitability and availability^62,63^, and the type, size and quality of a prey item^64^. This information reaches an individual down-current from the source as a cocktail of chemical cues that includes information that is ecologically relevant to a particular species, and potentially less relevant information from all the chemical activity within the community up-current. Alarm odours are included in this cocktail and the present research shows that the ecological relevance of the odour can be modified by the background chemistry of the environment to alter the information content of the odour. In this way, fundamental decisions that can influence risk and survival are being altered by environmental chemistry, which can occur at a scale greater than the local population.

Complex ecosystems, like rain forests or coral reefs, are biologically diverse and inherently patchy due to the processes that disrupt, replenish and maintain the communities^65^. Patchiness of the benthic components of an ecosystem is fundamental to community biodiversity and will lead to a chemically-rich environment^66^, particularly in aquatic ecosystems where chemicals can provide ecologically relevant information on scales up to many times greater than the home range of an organism^33^. Surprisingly, the present research suggests that the spatial scale of impact of environmental chemistry on the perception of alarm odours in our system is small, and possibly less than 2 metres. While there are few estimates of the active space for waterborne compounds, they are on the same scale as shown here. Turner and Montgomery^67^ found that refuge use by a snail (*Physa acuta*) returned to normal 1 m from a caged predatory fish (*Lepomis gibbosus*). Similarly, Weissburg and Beauvais^68^ found that predatory blue crabs (*Callinectes sapidus*) affected the behaviour of their mud crab prey (*Panopeus herbstii*) from 1.5 down to below 0.5 m. It is unclear whether this loss of the potency of the chemicals in these and our system is a function of dilution or a deactivation of the active components. The commonality of the small spatial scales of aversive chemistry across multiple, very different systems and cue identities suggests that the loss of perception may be attributable to the fundamental way that odorants are transported in seawater. At this relatively small spatial scale, topography and boundary layers play an important role in influencing the chemical connectivity of patches^69,70^. Water in a non-uniform, layered environment like a shallow coral reef is turbulent and results in considerable diffusion and concentration variance, not a smooth diffusive gradient^33,71^. Flow properties and boundary layer turbulence have been shown to have a major influence on the likelihood that an odour cue will be detected^69^, or chemistries may co-mingle between water bodies. Substantial research suggests that the small-scale fluid dynamics, particularly turbulent flows over complex reef surfaces, are critical to the interactions that occur among chemical components and the information aquatic organisms receive to inform behaviour (reviewed by^33^). This will be a useful line of research to further understand the spatial limits of the aversive chemistry that emanates from dead-degraded coral patches.

Although the present study found that the chemical effect of dead-degraded coral was potent at a small spatial scale, we also found it was reversible. Transferral of fish who had not been able to detect alarm odours on dead-degraded corals to live coral patches restored their ability to respond to alarm odours with an appropriate antipredator response. This occurred after a 20 to 40 min habituation period, so their ability to detect alarm odours may have been restored earlier. Current research suggests that environmental chemistry may modify the chemistry of the alarm odour rather than altering the sensory receptors of the fish^21^. Lönnstedt *et al.* ^18^ mixed a small aliquot (10 mL) of water that had passed over dead-degraded coral with the damage-released skin-extract (i.e.alarm odour) from conspecifics and found that this was sufficient to nullify the antipredator response in the Ambon damselfish tested within a 15 L tank of clean seawater. Ferrari *et al.* ^20^ also found that Ambon damselfish still responded to the damage-released odours from a rubble-dwelling congeneric, the blue-scribbled damsel (*Pomacentrus nagasakiensis*), in water from dead-degraded coral, suggesting that the olfactory receptors are unlikely to be altered by the chemicals from degraded corals. However, research on the olfactory receptor system of fishes suggests that there are over a hundred different types of receptor neuron types (of three main sorts: ciliated, microvillar and crypt cells) that are likely to have specific roles and receptivity^72^. Furthermore, limited research suggests that the alarm odour molecule may be complex (e.g.,^73^), and that they are likely to be discriminated using combinations of receptors to produce a complex signature^72^. It is therefore possible that chemicals within water from dead-degraded habitats are actively binding to the receptor sites and altering the way the alarm odour compound is perceived the olfactory system^72^, which are quickly flushed away when the water source changes. Further studies are required to determine the mechanism that underlies this chemo-behavioural interaction. If the detrimental effect on alarm odours is temporary as suggested in the present study, then anything that divorces the individual fish from the plume of active chemicals will aid the re-establishment of an effective alarm response.

The spatially and temporally discrete nature of the effect implies that the influence of environmental chemicals on the alarm odour response is dynamic and transient, as currents change and fishes move between patches. Because the movement of many juvenile fishes is very limited in the first few weeks to months on the reef^74^, the major force influencing the environmental chemicals to which they are exposed will be the tide. The tidal cycle, particularly around shallow topographically complex reefs, is a major driver of currents and often causes a predictable alteration, at times a reversal, in the direction of flow^70,75,76^. This may mean that, for a species resident on a live coral habitat patch, they may be influenced by the chemicals from a degraded neighbouring patch only at some states of the tide, depending upon the small-scale arrangement of non-coral habitats. Given the response to alarm odours is innate and central to the detection of risk^22,23^, this will mean that fishes will be exposed to potentially predictable temporal windows when they can detect risk using alarm odours, separated by periods when this is no longer possible. Experimental studies have shown that fishes use temporal patterns in alarm odours, or known risk cues, to modify their behaviour pattern such that they undertake the riskiest behaviours during the times of lowest risk^54,77–79^. Thus the temporal patterns of behaviour of site-attached fishes may be strongly influenced by predictable small-scale patterns in the current regime through their alteration of the local chemical environment, which includes alarm odours, cues from known predators and an array of chemical processes upcurrent. It is currently unknown whether fish may use other sources of information to inform risk, such as visual and mechanical cues (vibration and acoustic) to compensate for the lack of useful chemical information during periods when alarm odours are not detectable. Such sensory compensation^80^ has been found in situations when sediment^81^, light^61^ or topography^82^ has obscured visual cues. However, the lack of a visual predator cue can be related to a perceivable change in the photic environment and so easily gauged. In contrast, the olfactory system may not be able to disambiguate a lack of odour from the situation where there is no perception because of masking or modification, and there are currently no examples of sensory compensation for a lost olfactory sense. Clearly, further research is warranted that examines whether and how fishes compensate for temporal fluctuations in the loss of information from one sensory mode.

The present study determined that common algal components of dead-degraded coral had a nullifying effect on alarm odours for our damselfish. Note that, while almost all seawater controls showed some reduction in activity and feeding this is a typical feature of these laboratory experiments (e.g.,^20,55^). The slight reduction in all treatments is also likely due to the stress associated with the artificiality of the tank environment inducing a mild neophobia^28^. The key interpretation from this experiment comes from the comparison of the treatments with their saltwater controls and the resulting effect sizes. Unsurprisingly, the dead coral skeleton had no effect on alarm odour reactions, nor did the skeleton that had been scrubbed of the largest macroalgae. In contrast, water that had been in contact with two common components of the degraded substrata, the filamentous cyanobacteria *Okeania* sp. and the blooming diatom *Pseudo-nitzschia* sp.prevented the Ambon damselfish from mounting a typical alarm odour response. Members of the genus *Okeania* are known to produce toxic metabolites that inhibit or interfere with key biological processes, including the interference of signal transduction in sodium channels, altering signalling proteins, inducing programmed cell death, and inhibiting membrane transporters, receptors and topoisomerases^43^, the last of which is important in DNA replication. Similarly, while much less research has been conducted on the chemistry of diatoms than the chemically rich cyanobacteria, approximately half of the known species of *Pseudo-nitzschia* produce toxic components such as domoic acid, which is a potent neurotoxin^83^. It should be noted that while the algal components tested have been previously found to be chemically rich, the active components that lead to the alarm odour nullifying effect may originate from a companion organism, such as a bacteria, that commonly associates with the substratum types^84^. Our evidence suggests that the biologically active chemistry of these cyanobacteria and diatoms or their associated biota are in some way altering the fish-alarm odour interaction so that is no longer recognised as an indication of danger.

Interestingly, it was not only the components associated with degraded coral habitat that altered the alarm odour response in our damselfish, but also one of the two macroalgae that are common components of the algal community on coral reefs. While the ephemeral brown algae *Padina* had no effect, water that had been in contact with the red algae *Galaxaura* significantly altered the alarm odour response. Members of the genus *Galaxaura* have been found to produce allelochemicals that cause bleaching and suppress the photosynthetic efficiency of hard corals^85^. Given that *Galaxaura* is common on tropical reefs, where at times it can form large stands (Fig. 1), this finding has ramifications for the likely frequency with which environmental chemistry has the capacity to alter the efficacy of fish alarm odours. The finding also suggests that further screening of algae would be useful so that the spatial and temporal extent of the chemical interactions can be understood.

Large numbers of fish species associate with live coral during their early settled life^86^. At this time they are most vulnerable to predators, partly due to their poor understanding of the identity of local predators^48^. Jones *et al.* ^87^ found that while most species did not associate with live corals necessarily as adults, 65% of species used live coral as nursery habitat. Many of these are likely to be influenced by the modification of alarm odours from temporal patterns in the chemistry of the surrounding habitats (e.g.,^20^). Given the central role that alarm odours play in the detection, learning and quantification of risk^22,23,88^, understanding how the local benthic community and small-scale hydrology alters the chemical information used by fishes to inform choices will be key to determining how changes in coral cover will alter fish community dynamics.

### Ethics statement

All work carried herein was in accordance with the James Cook University Animal Ethics guidelines (JCU Animal Ethics approvals A2005 and A2080).
